# ATP-Dependent Infra-Slow (<0.1 Hz) Oscillations in Thalamic Networks

**DOI:** 10.1371/journal.pone.0004447

**Published:** 2009-02-12

**Authors:** Magor L. Lőrincz, Freya Geall, Ying Bao, Vincenzo Crunelli, Stuart W. Hughes

**Affiliations:** School of Biosciences, Cardiff University, Cardiff, United Kingdom; Julius-Maximilians-Universität Würzburg, Germany

## Abstract

An increasing number of EEG and resting state fMRI studies in both humans and animals indicate that spontaneous low frequency fluctuations in cerebral activity at <0.1 Hz (infra-slow oscillations, ISOs) represent a fundamental component of brain functioning, being known to correlate with faster neuronal ensemble oscillations, regulate behavioural performance and influence seizure susceptibility. Although these oscillations have been commonly indicated to involve the thalamus their basic cellular mechanisms remain poorly understood. Here we show that various nuclei in the dorsal thalamus *in vitro* can express a robust ISO at ∼0.005–0.1 Hz that is greatly facilitated by activating metabotropic glutamate receptors (mGluRs) and/or Ach receptors (AchRs). This ISO is a neuronal population phenomenon which modulates faster gap junction (GJ)-dependent network oscillations, and can underlie epileptic activity when AchRs or mGluRs are stimulated excessively. In individual thalamocortical neurons the ISO is primarily shaped by rhythmic, long-lasting hyperpolarizing potentials which reflect the activation of A1 receptors, by ATP-derived adenosine, and subsequent opening of Ba^2+^-sensitive K^+^ channels. We argue that this ISO has a likely non-neuronal origin and may contribute to shaping ISOs in the intact brain.

## Introduction

Infra-slow oscillations (ISOs) with a periodicity of tens of seconds to a few minutes are an important, but under-investigated, feature of macroscopic brain activity. Such oscillations were first observed in local field potential (LFP) recordings from the rabbit neocortex [1] but have since been observed in several other mammals [2]–[4] and are readily detectable in full band EEG (fbEEG) recordings from humans [5]. ISOs are also a consistent and important feature of the fMRI BOLD signal during the resting state, or so-called default mode, in humans [6]–[9] and in anaesthetized nonhuman primates [10] and rats [11]. The emerging functions of ISOs include a role in modulating gross neuronal excitability, being correlated with fluctuations in the amplitude of faster EEG oscillations in several well-defined frequency bands in the 1–80 Hz range [5], [9], and in regulating behavioural performance [12], [13]. They are also closely associated with several types of epileptic events [5], [14], [15]. For example, generalized polyspikes in patients with the catastrophic Lennox-Gastaut syndrome (LGS) occur significantly more frequently during the active phase of the so-called cyclic alternating pattern (CAP) [15], an ISO with a periodicity of ∼20–40 seconds that participates in the dynamic organization of non-rapid eye movement (NREM) sleep EEG architecture [16].

Although the origins of ISOs are not well understood, recent EEG and imaging studies in humans support a key involvement of subcortical structures and, in particular, the thalamus [5], [9], [17]. This is consistent with animal studies where ISOs in the thalamus have been directly observed in both anaesthetized [4], [18]–[21] and freely moving [3], [19] preparations and are evident in LFP [3], [4], single unit [18], [19] and intracellular [20] recordings. An ISO is also present in thalamic firing during the generation of so-called cyclic paroxysms [22]. Cyclic paroxysms are experimental electrographic seizures [22], [23] which recur with an infra-slow periodicity of ∼40–60 seconds, consist of a mixture of slow (∼2–4 Hz) spike/poly-spike wave (SW/PSW) complexes intermingled with fast (10–20 Hz) runs and which have been likened to the EEG activity that occurs during LGS in humans [24].

In this study we show that TC neurons in slices of various cat sensory thalamic nuclei maintained *in vitro* can generate a robust ISO at ∼0.005–0.1 Hz that primarily depends on A1 receptor-mediated signalling and, probably, opening of G protein-coupled inwardly rectifying K^+^ (GIRK) channels. This ISO is greatly facilitated by activation of mGluRs and/or AchRs and is a network phenomenon that is present in LFP recordings, modulates regional α oscillations and can be associated with cyclic paroxysmal episodes that possess identical features with those previously described *in vivo* [22], [23]. We suggest that this ISO has a probable non-neuronal origin and may make a contribution to the enigmatic ISOs that are a consistent and prominent feature of the fMRI BOLD signal during the resting state in humans and animals [6]–[9].

## Results

### Moderate activation of AchRs and/or mGluRs facilitates a population ISO in TC neurons of various sensory thalamic nuclei

**Figure 1:**
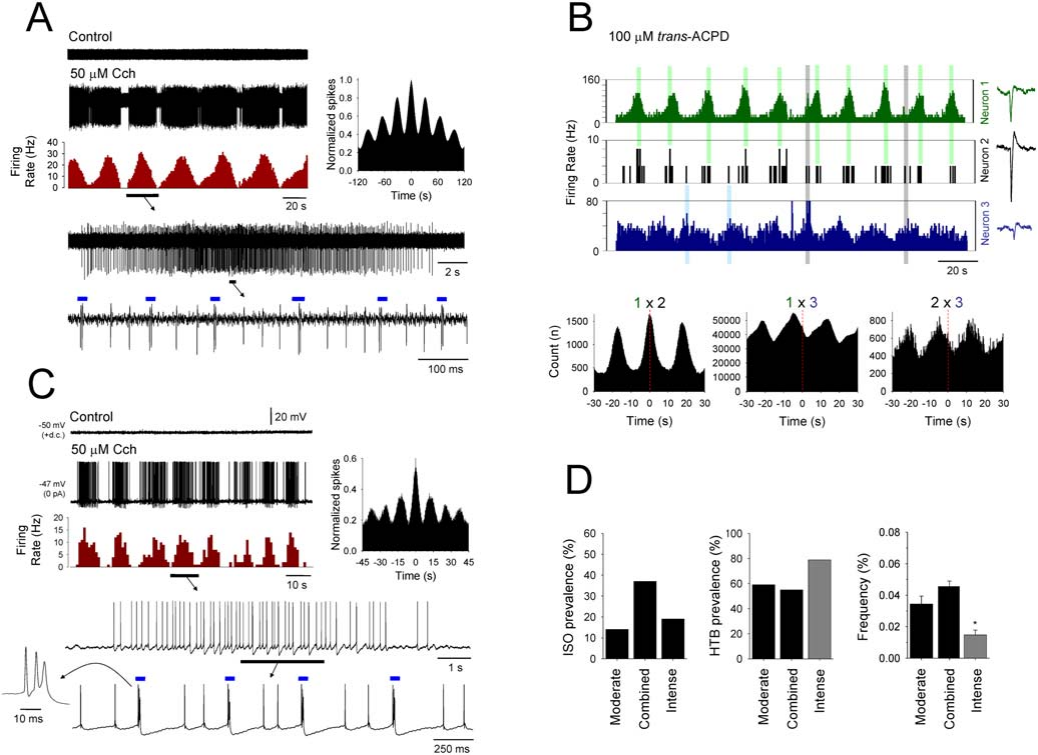
Appearance of a population ISO in TC neurons following moderate activation of AchRs and/or mGluRs. A. Application of of 50 µM Cch induces spontaneous firing in a TC neuron that is modulated by an ISO at ∼0.03 Hz (second trace from top). The corresponding auto-correlogram and firing rate histogram are shown to the right and immediately below, respectively. Shown further below are enlarged sections from one of the firing episodes, as indicated, which reveal a mixture of tonic firing and HT bursts (blue bars). B. Firing rate histograms from three distinct, simultaneously recorded VB TC neurons (respective spike waveforms are shown to the far right) showing an ISO at ∼0.05 Hz in the presence of 100 µM *trans*-ACPD (See also Fig. S1). Note how the firing of neuron 3 (bottom plot, blue bars) generally peaks before that of neuron 1 (top plot, red bars) but that neuron 2 (middle plot, grey bars) seems to be directly linked by both cells (light red and light blue bars). The plots below show the respective cross-correlograms for the 3 possible neuron-neuron combinations as indicated. C. Intracellular recording of an LGN TC neuron which exhibits an ISO at ∼0.075 Hz following 50 µM Cch application. The corresponding auto-correlogram and firing rate histogram are shown to the right and immediately below, respectively, and enlarged sections are shown further below, as indicated (HT bursts indicated by blue bars). D. Histograms summarizing the effect of moderate (100 µM *trans*-ACPD or 50 µM Cch), combined (100 µM *trans*-ACPD and 50 µM Cch) and intense (200 µM *trans*-ACPD or 100 µM Cch) agonist application on ISO prevalence (left), percentage of ISOs involving HT bursting (middle) and ISO frequency (right).

**Figure 2:**
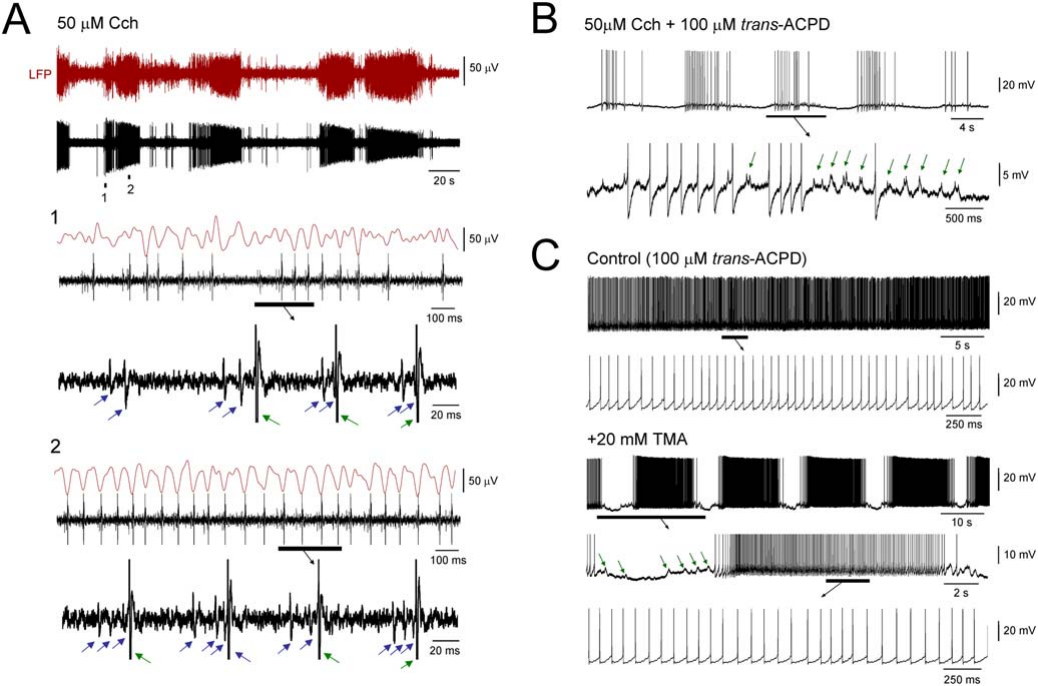
The ISO modulates network oscillations in the α (8–13 Hz) frequency band and can underlie cyclic paroxysms. A. Simultaneous LFP and multi-unit recording from the LGN in the presence of 50 µM Cch showing that the ISO in firing (bottom, MUA) is associated with a modulation of faster oscillations in the LFP (top). The sections labelled 1 and 2 are expanded below and reveal that the ∼13 Hz field oscillations are associated with HT bursts (blue arrows in expanded sections) that appear to drive activity in an additional tonic firing cell (red arrows in expanded sections) (see [27]). B. ISO at ∼0.1 Hz recorded intracellularly from an LGN TC neuron where the most depolarized phase of the oscillation is crowned not only by action potentials but also by a combination of spikelets and burstlets (green arrows in the enlarged section below). C. Top traces: LGN TC neuron recorded intracellularly showing continuous tonic firing (see enlarged section below). Bottom traces: application of 20 mM TMA brings about an ISO at ∼0.05 Hz. In this condition the cell also exhibits brief depolarizing events (green arrows) that occur just before and after the transient firing episodes (see enlargements as indicated).

Following the individual or combined application of moderate concentrations of the Group I/II mGluR agonist, *trans*-ACPD (100 µM) [25], [29] and/or the non-specific AchR agonist, carbachol (Cch) (50 µM) [27] we found that a subset of TC neurons (individual drug application: 14%, n = 27 of 192; combined drug application: 37%, n = 40 of 109) from the cat LGN, MGN and VB maintained *in vitro* exhibited spontaneous firing that was modulated by a prominent infra-slow (<0.1 Hz) oscillation (ISO) (individual drug application: 0.034±0.01 Hz; n = 11; combined drug application: 0.046±0.004 Hz; n = 20) (Fig. 1 and Figs. S1 and S2). This ISO was evident in both extracellular single unit and intracellular recordings and consisted of prolonged periods of waxing and waning action potential output (peak firing rate: 27.8±5.9 Hz; n = 11) that were usually separated by periods of quiescence (Fig. 1 and Figs. S1 and S2). This action potential output could comprise either episodes of tonic firing only (individual drug application: 41%, n = 11 of 27; combined drug application: 45%, n = 18 of 40) (Fig. S1A), episodes of intermingled tonic firing and high-threshold (HT) bursts (individual drug application: 48%, n = 13 of 27; combined drug application: 48%, n = 19 of 40) (Figs. 1A, 1C and Fig. S1C) or periods of HT bursts only (individual drug application: 11%, n = 3 of 27; combined drug application: 7%, n = 3 of 40) (Fig. S1B). In most instances, ISOs were highly rhythmic (e.g. Fig. 2B and 2C), but could occasionally exhibit a more irregular appearance (e.g. Fig. 2A). Once established ISOs were extremely robust and could last for several hours.

Multiple unit extracellular recordings revealed that ISOs were often simultaneously present in groups of closely situated neurons (n = 5) (Fig. 1B). In some of these recordings, we observed a clear delay of several seconds (5.7±1.9 s; n = 5 cell pairs) between the peak of firing in different cells suggesting a propagating ‘wave-like’ phenomenon within the slice and hinting at the presence of a spatially distributed ISO-generating mechanism. Importantly, intracellular recordings showed that ISOs *in vitro* are almost exclusively reliant on mGluR or AchR activation because manually depolarizing cells by injecting steady depolarizing current in the absence of trans-ACPD or Cch revealed an infra-slow modulation of firing in only 2 out of 110 of cases (data not illustrated) (see also [30]). In all cells tested, ISOs were resistant to the application of blockers of ionotropic glutamate, GABA_A_ and GABA_B_ receptors (CNQX or NBQX, 10–20 µM; APV, 100 µM; SR95531, 10 µM; CGP54626, 10 µM; n = 7) (Fig. S1B).

### The ISO modulates gap junction (GJ)-dependent network oscillations in the α (8–13 Hz) band

In some extracellular recordings, discrete ISO-derived epochs of HT bursting appeared to drive synchronous firing in additional cells, and could be associated with recurrent LFP oscillations in the α (8–13 Hz) band (frequency: 11.3±0.6 Hz; peak-to-peak amplitude: 79.2±4.8 µV; n = 5) (Fig. 2A) [25], [27], [29]. Consistent with this, we obtained intracellular recordings of TC neurons (n = 8) that displayed unambiguous rhythmic spikelets (amplitude: 2.1±0.04 mV; time to peak: 1.9±0.05 ms; duration: 8.8±0.07 ms; n = 20 events) and burstlets (i.e. groups of spikelets which represent HT bursts that have been communicated via GJ coupling) that showed a clear infra-slow cyclic modulation (Fig. 2B and Fig. S3A). This indicated that ISOs can influence the generation of faster GJ-dependent network oscillations [25], [29] and that, at least in some TC neurons, the expression of the ISO involves GJ signalling. In support of this, in a small number of extracellular single unit recordings (33%, n = 2 of 6) the ISO was abolished by the putative GJ blocker, 18β-glycyrrhetinic acid (18β-GA) (100 µM) (Fig. S3B), whereas the glycyrrhetinic acid derivative that is inactive as a GJ blocker, glyzyrrhizic acid (GA) (100 µM) failed to affect the ISO in any cells (n = 7) (Fig. S3C). We also found that in 3 of 9 (33%) extracellular and 2 of 8 (25%) intracellular recordings the putative GJ opener trimethylamine (TMA) was able to reversibly bring about an ISO in TC neurons where it was not originally present (frequency: 0.03±0.007 Hz; n = 5) (Fig. 2C and Fig. S3D).

### ISOs induced by a more intense activation of AchRs or mGluRs can be associated with cyclic paroxysms

**Figure 3:**
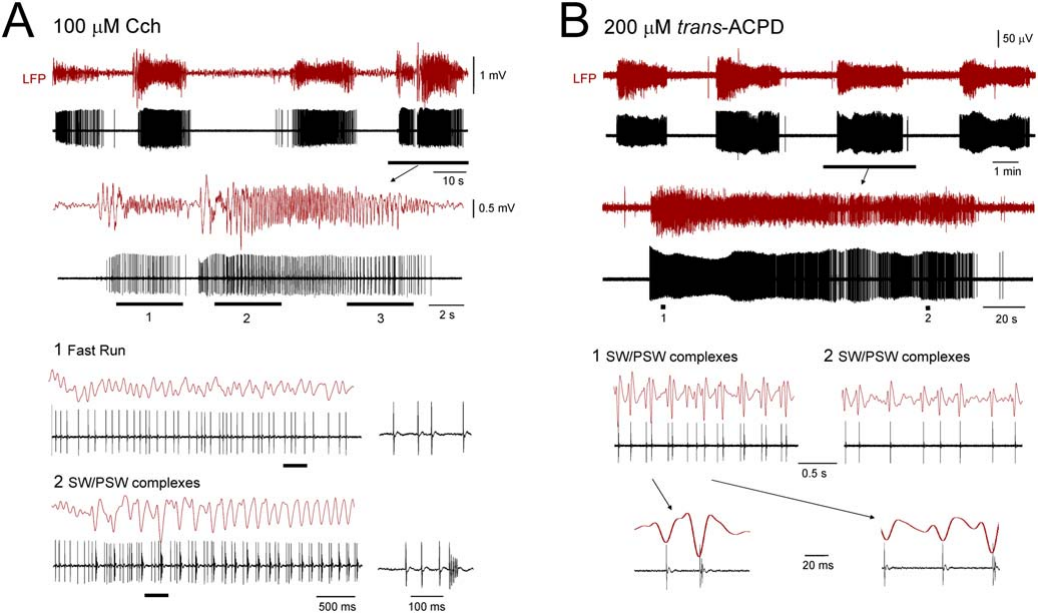
Following intense activation of AchRs or mGluRs the ISO can be associated with cyclic paroxysms. A. Simultaneous LFP and unit recording from the VB in the presence of 100 µM Cch showing an ISO at ∼0.03 Hz. The underlined section is enlarged below. Additional enlargements of the sections marked 1 and 2 are shown further below, as indicated, and reveal that LFP activity consists of a mixture of fast runs at ∼10 Hz corresponding to periods of tonic firing (1), and rhythmic SW/PSW complexes at ∼4 Hz (2) which are related to unusually powerful HT burst activity. In 1 and 2, the underlined sections are expanded to the immediate right. B. Simultaneous LFP and unit recording from the LGN in the presence of 200 µM *trans*-ACPD showing an ISO at ∼0.004 Hz. The underlined section is enlarged below. Additional enlargements of the sections marked 1 and 2 are shown further below, as indicated, and reveal that field activity consists of rhythmic SW/PSW complexes at ∼3 Hz which are closely related to HT burst activity in the simultaneously recorded TC neuron.

When applied individually, increasing the concentration of either *trans*-ACPD or Cch (to 200 µM and 100 µM, respectively) led to a slight increase in the percentage of cells showing ISOs (19%, n = 19 of 98; 200 µM *trans*-ACPD: 17%, n = 10 of 56; 100 µM Cch: 21%, n = 9 of 42) (Fig. 1D and Fig. S2). However, the mean frequency of these oscillations was significantly lower (0.015±0.003 Hz; range 0.004–0.04 Hz, n = 16; p<0.01) than during moderate application (Fig. 1D). Furthermore, following this more intense mGluR or AchR activation, recurrent episodes of firing were of a higher mean frequency (peak firing rate: 39.8.7±3.8 Hz; p<0.01; n = 12) and more commonly involved HT bursting (79%, n = 15 of 19) (Fig. 1D). Moreover, in this condition, these HT bursts were considerably more powerful (mean spikes per burst: 3.7±0.3 *vs* 2.2±0.1; p<0.01; n = 40 events) (Fig. 3A) and rather than being correlated with normal α rhythms, could be associated with cyclic paroxysmal activity [22], [23] in the LFP comprising recurring sequences of rhythmic spike wave (SW) and poly-spike wave (PSW) complexes at ∼2–4 Hz (mean frequency: 2.9±0.3 Hz; peak-to-peak amplitude: 268.2±85.0 µV; n = 5) that were sometimes intermingled with fast runs at ∼10–20 Hz (mean frequency: 14.1±3.8 Hz; peak-to-peak amplitude: 114.0±58.2 µV; n = 3) (Fig. 3A and B).

### The ISO in individual neurons is primarily shaped by long-lasting rhythmic hyperpolarizing potentials

**Figure 4:**
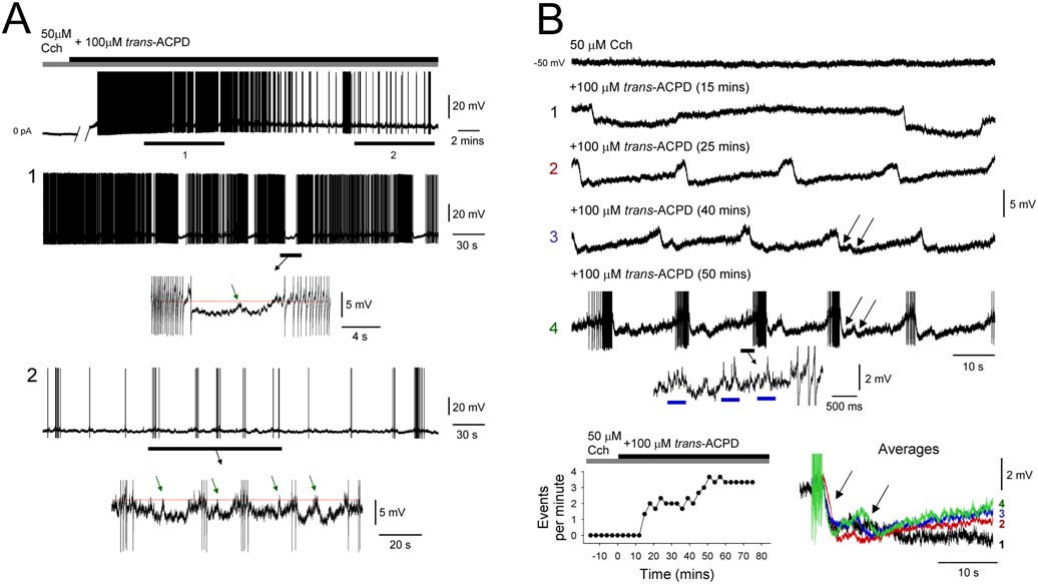
Progressive development of the ISO from a state of quiescence in individual TC neurons is characterised by the appearance of long-lasting rhythmic hyperpolarizing potentials. A. Intracellular recording of a TC neuron in the presence of 50 µM Cch which does not exhibit spontaneous firing (top trace, left). Following the additional application of 100 µM *trans*-ACPD, the cell first exhibits continuous firing before eventually exhibiting an ISO at ∼0.025 Hz (top trace, right). The sections labelled 1 and 2 are expanded below and show how the ISO develops as a progressive increase in the frequency of transient hyperpolarizing voltage excursions, and corresponding pauses in firing. In both 1 and 2, the underlined sections are expanded below and show the consistent presence of brief depolarizing events during the transient hyperpolarizing excursions (green arrows). B. Intracellular recording of a TC neuron in the presence of 50 µM Cch which does not exhibit spontaneous firing (top trace). Following the additional application of 100 µM *trans*-ACPD the cell exhibits stereotypical long-lasting hyperpolarizing potentials which gradually increase in frequency (1–3) until an ISO with a stable frequency of ∼0.05 Hz is established (4, action potentials truncated). The underlined section in 4 is enlarged below and shows groups of spikelets (blue bars). The plot in the bottom left corner shows the time-course of the increase in the frequency of these hyperpolarizing potentials with *trans*-ACPD application. The bottom right panel shows averages of the hyperpolarizing potentials at different stages of *trans*-ACPD application, as indicated. Note the complex biphasic nature of these events (see arrows indicating initiation of each phase).

**Figure 5:**
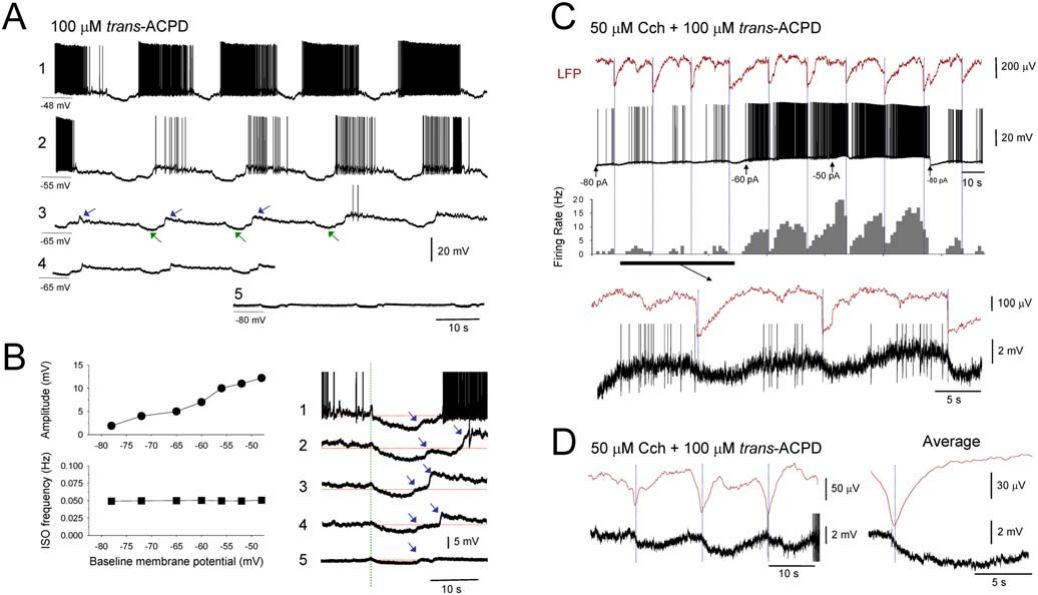
Response of the ISO to changes in membrane polarization and the presence of the ISO in LFP recordings. A. Intracellular recording of an ISO in a TC neuron in the LGN at different levels of steady current. Note that although ISO is mainly sculpted by long-lasting rhythmic hyperpolarizing potentials (green arrows in 3) it also involves depolarizing events (blue arrows in 3) (See also Figs. S5B and S5C). B. Plots showing that the amplitude (top) of the long-lasting hyperpolarizing potentials exhibited by the neuron in A is reduced as the cell is hyperpolarized but that the frequency (bottom) of the ISO is unaltered at ∼0.05 Hz. The traces to the right show enlarged examples of the long-lasting hyperpolarizing potentials in A (aligned at their start by the green dotted line at different levels of membrane polarization, red dotted line indicates the baseline membrane potential; numbers correspond to the traces in A). Note again the presence of additional depolarizing events (blue arrows). C. Simultaneous LFP and intracellular TC neuron recording of an ISO at ∼0.06 Hz (dark red trace) in the MGN (black trace) at different levels of steady injected current as indicated. The corresponding firing rate histogram is shown immediately below. Shown further below is an enlarged section of the recording (as indicated, action potentials truncated) illustrating that the negative peaks of the LFP (blue vertical lines) are coincident with a hyperpolarization and an accompanying suppression in firing in the TC neuron. B. Additional simultaneous LFP and intracellular TC neuron recording of an ISO at ∼0.083 Hz (red trace) obtained in the LGN. The traces to the right show the LFP negative peak-triggered averages for the LFP and membrane potential. (10 µM SR95531 and 10 µM CGP54626 were present for the recording shown in C).

In order to better understand the cellular events underlying the ISO, we closely examined the temporal development of this phenomenon from a state of quiescence following moderate *trans*-ACPD and/or Cch application. In all cases, the emergence of the ISO was associated with a progressive increase in the occurrence and rhythmicity of a long-lasting hyperpolarizing potentials (amplitude at −60 mV: 6.9±1.0 mV; duration: 15.8±1.3 s; n = 16) until a stable ISO was established (Fig. 4A and B). In most cells, these potentials were highly conserved from cycle to cycle, often displayed a prominent biphasic waveform (time between the onset of the two phases: 4.1±0.09 s; n = 5) (Fig. 4B, see arrows; see also Fig. S4B) and were similar to those we have shown previously to occur in a small number (∼3%) of TC neurons in control conditions [30]. We also often observed additional relatively faster depolarizing events (amplitude: 4.3±0.4 mV; duration: 0.9±0.1 s; n = 20 events) that mainly occurred either just prior to the onset of the long-lasting hyperpolarizing potentials (Figs. 5A and B and Fig. S5B) or during their repolarizing phase (Fig. 2C and Fig. S5C). Thus, the ISO in individual TC neurons is fundamentally shaped by long-lasting rhythmic hyperpolarizing potentials but also involves relatively faster depolarizing events.

The frequency of long-lasting hyperpolarizing potentials (and therefore the ISO) was unaffected by varying the level of steady injected current (Figs. 5A and B). However, the amplitude of these events was reduced as TC neurons were hyperpolarized, becoming indiscernible at ∼−85 mV (n = 5) (Fig. 5B). Combined with the finding that the ISO can be simultaneously present in distinct neurons (i.e. Fig. 1B), the invariance of ISO frequency in individual neurons with respect to alterations in steady current strongly suggested that it is a network rather than an intrinsic phenomenon. As direct confirmation of this we noted that ISOs could also be directly identified as infra-slow fluctuations in the LFP (mean peak-to-peak amplitude: 74.4±12.9 µV; n = 16) (Figs. 5C and D). Interestingly, simultaneous recordings of the ISO in the LFP with the subthreshold activity of individual TC neurons revealed that the ‘sharp’ negative field deflections were always coincident with the onset of the long-lasting hyperpolarizing potentials (n = 8) (Figs. 5C and D). Thus, during spontaneous action potential output, the firing of cells was suppressed at the time of the negative LFP peak (Fig. 5C).

### The long-lasting rhythmic hyperpolarizing potentials are dependent on A1 receptor signalling

**Figure 6:**
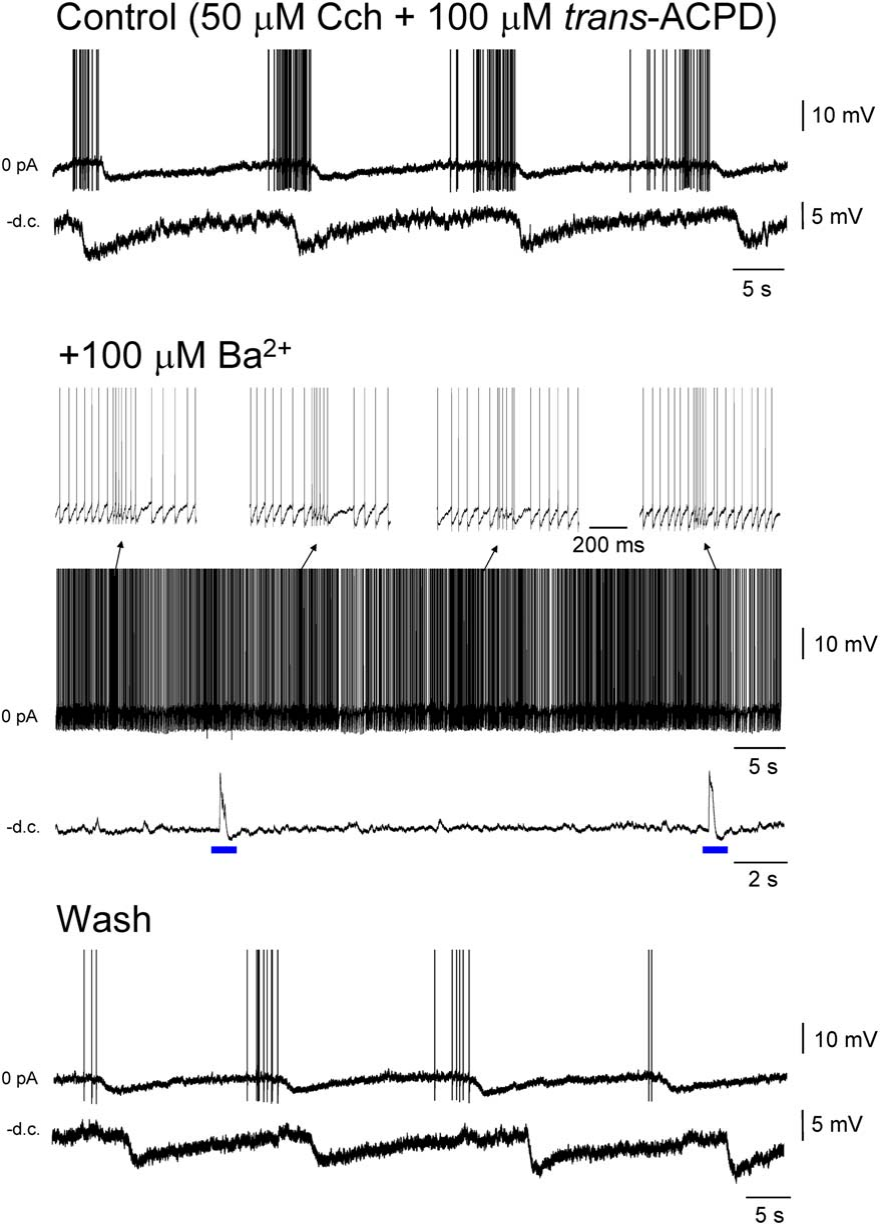
The long-lasting rhythmic hyperpolarizing potentials are blocked by Ba^2+^. Top panel: ISO recorded intracellularly in the LGN in the absence of steady injected current (top) and following the injection of a small amount of steady hyperpolarizing current (bottom). Middle panel: 100 µM Ba^2+^ abolishes the long-lasting rhythmic hyperpolarizing potentials that constitute the ISO. In the absence of steady inject current the neuron now exhibits continuous firing that is occasionally subject to short lasting (∼0.2–1 s) increases whereas when the cell is hyperpolarized it exhibits occasional brief depolarizing events (blue bars, average of five events shown below). Bottom panel: following washout of Ba^2+^, the neuron reverts to its original activity. (10 µM CNQX and 100 µM APV were present during this experiment and action potentials have been truncated in all panels).

In agreement with the finding that ISOs are resistant to ionotropic glutamate, GABA_A_ and GABA_B_ receptor blockers, the ISO-related long-lasting hyperpolarizing potentials were resistant to the combined application of the GABA_A_ and GABA_B_ receptor antagonists, SR95531 (10 µM) and CGP54626 (10 µM), respectively (n = 5) (e.g. Fig. 5C). This is consistent with previous work showing a resistance of these events to bicuculline methiodide [30], a study which additionally demonstrates that they do not involve small conductance Ca^2+^-activated K^+^ channels [31]. Long-lasting hyperpolarizing potentials were also resistant to blockade of Na^+^ channels with tetrodotoxin (TTX) (1 µM) (n = 6) and unaltered by the combined application of the respective AMPA/kainate and NMDA receptor antagonists, CNQX (or NBQX) (10–20 µM) and APV (100 µM) (n = 6) (Figs. S4B, S5A, S5B and S5C), which also failed to affect the faster depolarizing events. However, consistent with their disappearance close to the K^+^ equilibrium potential (see above) (Figs. 5A and B), long-lasting hyperpolarizing potentials were reversibly abolished in all cases by Ba^2+^ (100 µM) (n = 4) (Fig. 6). This facilitated the expression of faster depolarizing events, thereby transforming the suprathreshold manifestation of the ISO from firing that is rhythmically interrupted by prolonged periods of suppression to firing that showed rhythmically recurring, relatively brief (0.2–1 s) increases (Fig. 6, middle panel).

**Figure 7:**
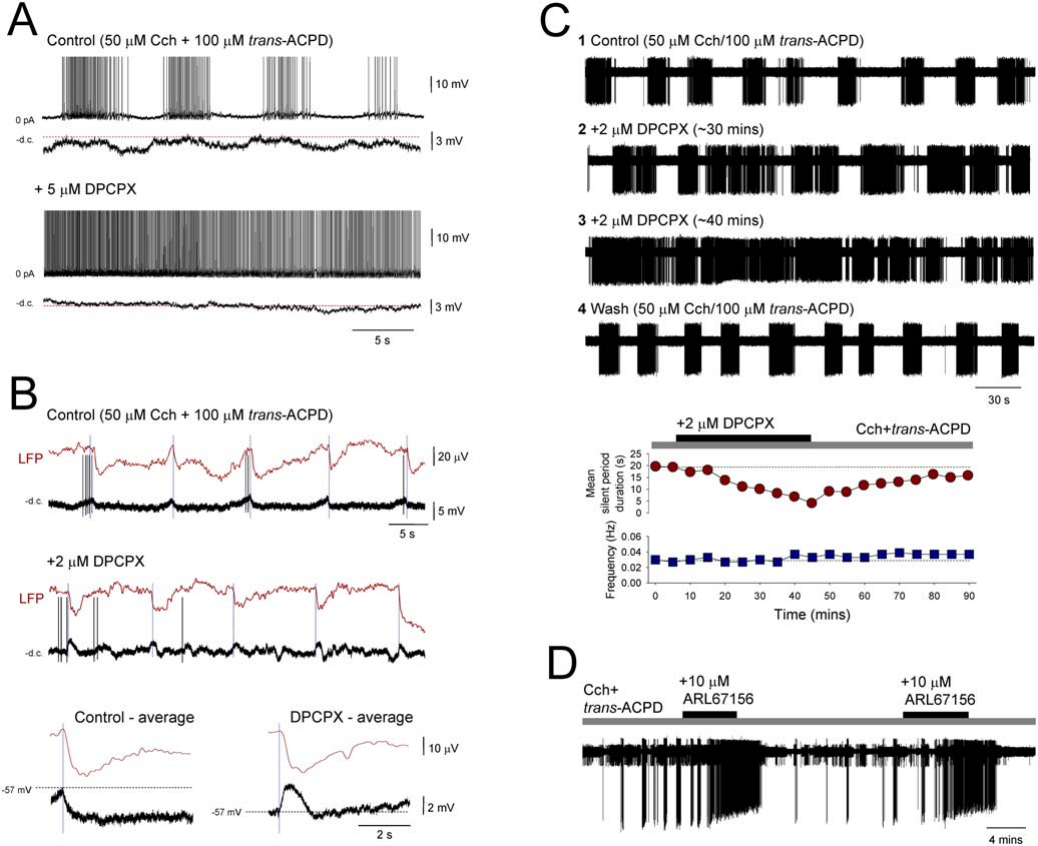
The long-lasting rhythmic hyperpolarizing potentials reflect activation of A1 receptors by ATP-derived adenosine. A. Complete block of the ISO in an LGN TC neuron following application of the A1 receptor antagonist DPCPX (5 µM). B. Top panel: simultaneous LFP and intracellular TC neuron recording of the ISO in the LGN. Below: DPCPX disrupts the expression of the ISO in the intracellular recording but not in the LFP. This is more clearly illustrated by the membrane potential averages shown below which reveal that the long-lasting hyperpolarizing potentials observed prior to DPCPX application (left) are abolished and replaced by a shorter lasting (∼1 s) depolarizing event (right). C. Plots showing the effect of DPCPX on the mean duration of the silent period (top) and the overall frequency (bottom) of an ISO observed with an extracellular single unit recording in the VB in the presence of 50 µM Cch. D. Extracellular single unit recording of a VB TC neuron exhibiting the ISO in the presence of 50 µM Cch. The ecto-ATPase inhibitor ARL67156 reversibly converts the ISO into continuous firing. (action potentials have been truncated in A and B).

At the concentration used (i.e. 100 µM), Ba^2+^ preferentially blocks G protein-coupled inwardly rectifying K^+^ (GIRK) channels [32] suggesting that long-lasting hyperpolarizing potentials might be due to the opening of these channels via the phasic activation of receptors on TC neurons to which they are positively coupled. Apart from GABA_B_ receptors [33], the other main receptor type that is known to be positively coupled to GIRK channels in TC neurons is the adenosine A1 receptor [34]. We therefore tested the effect of the A1 receptor antagonist, DPCPX (2–5 µM) on the generation of ISO-related long-lasting hyperpolarizing potentials. DPCPX abolished these potentials in all cells tested (n = 5) (Fig. 7A and B). Furthermore, and consistent with the effects of Ba^2+^, this facilitated the expression of depolarizing events (Fig. 7B). Interestingly, DPCPX did not however, affect the generation of the ISO in the LFP (n = 5) (Fig. 7B). In agreement with its effect in intracellular recordings, in all cases, DPCPX (2–5 µM) caused a significant shortening of the quiescent period of the ISO, as observed with single unit extracellular recordings (% of control 22.3±5.0; n = 5), without altering its overall frequency (n = 5) (Fig. 7C).

An appealing possibility is that the A1 receptor-dependent slow rhythmic hyperpolarizing potentials reflect the release of ATP from glial cells and its subsequent breakdown to adenosine. In the retina, for example, ATP released from Müller cells degrades to adenosine and generates a slow postsynaptic inhibition of neurons through the activation of A1 receptors and opening of Ba^2+^-sensitive K^+^ channels [35] that is comparable to that shown here. To test whether a similar breakdown of ATP is responsible for shaping the thalamic ISO described in this study we examined the effect of the ecto-ATPase inhibitor, ARL 67156 (10 µM), on the extracellularly-recorded ISO. In all cases, ARL 67156 reversibly converted the ISO to continuous firing (n = 7) (Fig. 7D) indicating that the inhibitory phases of the ISO are due to the effects of ATP-derived adenosine.

## Discussion

We have shown that TC neurons in slices of the cat LGN, MGN and VB can exhibit a pronounced population ISO at ∼0.005–0.1 Hz which is greatly facilitated by mGluR and/or AchR activation, can modulate faster GJ-dependent network oscillations at α (8–13 Hz) frequencies [25], [27] and can be manifested as cyclic paroxysmal episodes when mGluRs or AchRs are stimulated excessively. In individual neurons the ISO is fundamentally shaped by long-lasting rhythmic hyperpolarizing potentials which reflect the activation of A1 receptors by ATP-derived adenosine and subsequent opening of Ba^2+^-sensitive K^+^ channels.

### Likely non-neuronal origin of the ISO

In the thalamus, the chief candidates for releasing ATP are astrocytes. Thalamic astrocytes exhibit spontaneous intracellular Ca^2+^ oscillations *in situ* [36] which can be highly rhythmic [37] and which occur in an almost identical range of frequencies (0.003–0.1 Hz) to the neuronal ISOs shown here. They are responsive to the activation of mGluRs [36] and AchRs (H.R. Parri, personal communication), potentially explaining the facilitatory effect of mGluR and AchR activation in this study, whereas the presence of a slow ‘wave-like’ co-activation of different neurons (i.e. Fig. 1B) and the sometimes biphasic nature of the long-lasting hyperpolarizing potentials fits well with the presence of slowly propagating Ca^2+^ waves in groups of thalamic astrocytes [36]. Interestingly, a link between ATP and low frequency oscillatory activity has also been demonstrated in the entorhinal cortex [38]. With regard to the faster depolarizing events which are also a component of the ISO, we suggest that whilst not appearing to involve glutamate, they may well be generated by one of the several other transmitters released by glial cells [39].

### Relationship to *in vivo* electrophysiological data

Several *in vivo* studies have shown the presence of ISOs in different sensory nuclei of the thalamus [3], [4], [18]–[21]. For example, in the rat LGN, an ISO at ∼0.01 Hz is present in unit firing and can be observed in both freely moving and anaesthetized animals [18], [19]. In similarity to the ISO described here, this oscillation usually consists of episodes of firing interspersed with periods of quiescence and is resistant to the antagonism of ionotropic glutamate and GABA receptors [19]. In the cat MGN, ISOs in the range 0.1–0.25 Hz have been directly observed with intracellular recording [20], with the appearance of these oscillations also being similar to those described here. ISOs at ∼0.02–0.3 Hz have also been observed in LFP recordings from the rat LGN [3], [21] and MGN [4], indicating that infra-slow activity in these structures can occur as more than simply single cell oscillations. Taken together, these studies show that ISOs are an integral component of thalamic activity, a view that is further supported by our demonstration that thalamic nuclei can autonomously generate population ISOs *in vitro*.

### Involvement of thalamic GJs in distributing the ISO

In some TC neurons the expression of the ISO depends on GJs. This is not only supported by the abolition of the ISO in a subset of extracellular recordings by putative pharmacological GJ blockade but also by the presence of unambiguous spikelets and burstlets in intracellular recordings that are rhythmically modulated on an infra-slow timescale. The presence of these rhythmically modulated spikelets and burstlets fits well with our finding that ISO-modulated HT bursting neurons appear to drive additional cells during α wave epochs and shows that the ongoing infra-slow modulation of α activity that is commonly observed *in vivo* [9], [40]–[47] can also be a feature of these oscillations in the isolated thalamus *in vitro* [25], [27], [48]. Whilst we cannot completely discount a possible contribution of GJs between non-neuronal cells in these phenomena, these findings overwhelmingly endorse previous suggestions that GJs between TC neurons are an important determinant of local thalamic network activity [25], [27], [49], [50].

### Local generation of cyclic paroxysms in the thalamus

That intense activation of either mGluRs or AchRs leads to cyclic paroxysms in isolated thalamic slices is notable because previous *in vivo* studies have suggested that such activity is generated solely in the neocortex and then spreads to the thalamus [22], [23]. Thus, the thalamus may play a considerably more active role in generating these paroxysms than had previously been thought. An additional important observation from the current study is that the type of bursting that is associated with SW/PSW complexes during paroxysmal activity *in vitro* is not generated by a conventional LTCP-mediated mechanism [51]–[53] but by a HT burst-generating mechanism [25], [27]. This is consistent with *in vivo* recordings where the TC neuron bursting associated with SW/PSW complexes appears to be incompatible with an LTCP-mediated origin, due to the presence of interspike intervals that are patently too large (>10 ms) for LTCP bursts (see especially Fig. 4 in [23] and Fig 7 in [22]), but is entirely reconcilable with an HT burst basis [25].

During paroxysmal activity HT bursts were often noted to be unusually powerful (e.g. Fig. 3A) compared to those shown to occur under more moderate receptor activation and to be related to normal α rhythms [25], [27], [29]. Such bursts are likely to be associated with a significant depolarization of TC neuron dendrites [25], [54], [55] and may consequently be related to an elevated and detrimental influx of Ca^2+^. Since *in vitro* cyclic paroxysms arise from an excessive activation of mGluRs or mAchRs and are related to the augmented and abnormal expression of HT bursting in TC neurons, it is reasonable to view them as a pathological extension of physiological α activity [25], [27], [48]. This is interesting because in humans, susceptibility to several types of seizures is enhanced during relaxed wakefulness and in the case of the Rolandic μ rhythm, the equivalent of the classical α rhythm in the somatosensory system, the distinction between a purely physiological rhythm and one that is overtly pathological is not easy to define [55]. Thus, in the whole brain, enhancements in the basic excitatory tone needed to sustain intermittent physiological α rhythms may be a key component in shifting the balance from normal brain activity toward seizure generation. Furthermore, the excessive Ca^2+^ entry associated with the resultant aberrant neuronal bursting might play a central role in promoting the catastrophic cellular damage that occurs in certain types of malignant epilepsies such as LGS.

### Functional significance

Although the presence of ISOs in the mammalian brain was first highlighted over 50 years ago [1], until relatively recently the importance of these oscillations has been largely ignored. This has mainly been due to the inability of conventional EEG apparatus to detect such slow fluctuations which instead requires the use of fbEEG or direct current recordings [5], [12], [57]. However, the finding from several independent studies that spontaneous oscillations at <0.1 Hz are a consistent and prominent feature of the fMRI BOLD signal during the resting state, or so-called default mode, of the human [8] and animal [10], [11] brain has led to a re-emergence of interest in ISOs. In humans these oscillations identify highly specific functional anatomical networks (termed resting state networks, RSNs) [6], [7], [9], some of which are thought to involve a significant contribution from the thalamus [9], [17]. Although there is considerable debate as to what extent such infra-slow cerebral fluctuations are related to infra-slow neuronal activity, it has become clear that they at least correlate closely with episodes of faster EEG oscillations in several well-defined frequency bands [9], including the α band [9], [44]–[46]. With respect to this, our study is the first to show that neuronal populations in the isolated thalamus, a brain area which is an integral component in several RSNs, exhibit prominent ISOs in the same frequency range as those observed in baseline fMRI signals, and which are correlated with faster network oscillations in the α band in a similar way to that observed in the whole brain.

## Materials and Methods

All procedures were carried out in accordance with local ethical committee guidelines and the U.K. Animals (Scientific Procedure) Act, 1986. All efforts were made to minimize the suffering and number of animals used in each experiment.

### Slice preparation and maintenance

Young adult cats (1–1.5 kg) were deeply anaesthetized with a mixture of O_2_ and NO_2_ (2∶1) and 2.5% isoflurane, a wide craniotomy performed and the brain removed. Sagittal slices (450–500 µm) of the dorsal lateral geniculate nucleus (LGN), the medial geniculate nucleus (MGN) and the ventrobasal complex (VB) were prepared and maintained as described previously [25]–[27]. For recording, slices were perfused with a warmed (35±1°C) continuously oxygenated (95% O_2_, 5% CO_2_) artificial cerebrospinal fluid (ACSF) containing (mM): NaCl (134); KCl (2); KH_2_PO_4_ (1.25); MgSO_4_ (1); CaCl_2_ (2); NaHCO_3_ (16); glucose (10).

### Sources of drugs

DL-2-amino-5-phosphonovaleric acid (APV), 6-*N*, *N*-diethyl-β-γ-dibromomethylene-D-adenosine-5′-triphosphate trisodium salt (ARL 67156), [S-(R*,R*)]-[3-[[1-(3,4-dichlorophenyl)ethyl]amino]-2-hydroxypropyl] (cyclohexylmethyl) phosphinic acid (CGP 54626), 6-cyano-7-nitroquinoxaline-2,3-dione (CNQX), 2,3-dihydroxy-6-nitro-7-sulfamoyl-benzo[f]quinoxaline-2,3-dione, (NBQX), 6-imino-3-(4-methoxyphenyl)-1(6*H*)-pyridazinebutanoic acid hydrobromide (SR95531) from Tocris-Cookson (UK); carbamylcholine chloride (carbachol, Cch), 18β-glycyrrhetinic acid (18β-GA), glycyrrhizic acid (GZA), trimethylamine (TMA) were obtained from Sigma (UK). All drugs were dissolved in ACSF except CGP 54626, DPCPX, 18β-GA and GZA which were dissolved in DMSO and then added to ACSF such that the total final volume of DMSO did not exceed 0.1%. 0.1% DMSO applied alone had no effect on LFP, extracellular and intracellular recordings.

### Electrophysiology and data analysis

Extracellular recordings were performed using glass pipettes filled with 0.5 M NaCl (resistance: 1–5 MΩ) connected to a Neurolog 104 differential amplifier (Digitimer Ltd., Welwyn Garden City, UK). LFP and unit activities were obtained by bandpass filtering at <20 Hz and 0.2–20 kHz, respectively. Independently mounted intracellular recordings, using the current clamp technique, were performed with standard-wall glass microelectrodes filled with 1 M potassium acetate (resistance: 80–120 MΩ), and in some cases 2% biocytin or neurobiotin, and connected to an Axoclamp-2A amplifier (Axon Instruments, Foster City, CA, USA) operating in bridge mode. All TC neurons were recorded from either lamina A or A1 of the LGN, the dorsal subdivision of the MGN or the ventral posterolateral (VPL) nucleus of the VB [26]. Impaled cells were identified as TC neurons using established criteria [26], [28]. Voltage and current records were digitally acquired and processed using pClamp 10 (Molecular Devices Corporation, Sunnyvale, CA, USA). Action potential firing rate histograms and autocorrelograms were generated using custom written transform routines in SigmaPlot 9 (Systat, Hounslow, UK). Statistical significance was assessed using Student's t-test. All quantitative data are expressed as mean±s.e.m.

## Supporting Information

Figure S1 Additional examples of the ISO recorded in the presence of 100 µM *trans*-ACPD. A. Extracellular recording in a cat LGN slice showing a lack of activity in control conditions (top trace). Application of 100 µM *trans*-ACPD induces spontaneous firing in a TC neuron that is modulated by an ISO at ∼0.025 Hz (second trace from top). The corresponding firing rate histogram is shown immediately below. Shown further below is an enlarged section from one of the firing episodes, as indicated, which consists of single spike activity only. The corresponding auto-correlogram is shown to the right. B. ISO at ∼0.035 Hz recorded from a TC neuron in a cat VB slice in the presence 100 µM *trans*-ACPD, 10 µM CNQX, 100 µM APV, 10 µM SR95531 and 10 µM CGP54626 showing episodes of waxing and waning HT bursting. Again, the corresponding firing rate histogram is shown immediately below and an enlarged section of HT bursting shown further below as indicated. The corresponding auto-correlogram is shown to the right. C. Intracellular recording of an LGN TC neuron in the presence of 100 µM *trans*-ACPD exhibiting an ISO at ∼0.075 Hz. The underlined section is enlarged below. Shown further below are additional enlarged sections (1 and 2) which reveal a mixture of tonic firing and HT bursts (blue bars) (cf. Fig. 1A and C).
(2.13 MB TIF)

Figure S2 Additional examples of the ISO following intense activation of AchRs or mGluRs. A. Top: irregular, continuous tonic firing recorded in an LGN TC neuron in the presence of 50 µM Cch (see underlined section enlarged below). Bottom: increasing the concentration of Cch to 100 µM converts this activity into an ISO at ∼0.006 Hz which includes periods of HT bursting (see underlined section is enlarged below). Furthermore, these bursts now appear to occasionally drive synchronous activity in additional cells (see blue arrows in bottom traces). B. ISO at ∼0.015 Hz recorded extracellularly from a TC neuron in the LGN in the presence of 200 µM *trans*-ACPD (note the presence of an additional smaller unit which does not exhibit an ISO). The corresponding firing rate histogram is shown immediately below. Shown further below is an enlarged section from one of the firing episodes, as indicated, which consists of single spike activity only. C. ISO at ∼0.025 Hz also recorded extracellularly from a TC neuron in the LGN in the presence of 200 µM *trans*-ACPD. The corresponding firing rate histogram is shown immediately below. Shown further below is an enlarged section from one of the firing episodes, as indicated, which in this case comprises HT bursting.
(1.66 MB TIF)

Figure S3 Additional evidence of a role for GJs in the manifestation of the ISO in individual TC neurons. A. Histogram showing the frequency of burstlets over a 14 minute period in an LGN TC neuron recorded intracellularly. Note how this frequency is modulated by an ISO with a frequency of ∼0.006 Hz (blue vertical lines indicate the points of minimum interburstlet frequency). Interestingly, this cell did not otherwise show any overt infra-slow changes in baseline membrane potential. The inset above shows three consecutive burstlets recorded at the point indicated by the arrow. B. ISO at ∼0.006 Hz recorded extracellularly in a VB TC neuron is abolished by application of the GJ blocker, 18β-GA. C. ISO at ∼0.02 Hz recorded extracellularly from an LGN TC neuron in the presence of 100 µM *trans*-ACPD (top) is unaffected by GZA application (bottom). D. Top traces: LGN TC neuron recorded extracellularly showing continuous tonic firing (see enlarged section below). Middle traces: following addition of 20 mM TMA, the neuron exhibits an ISO at ∼0.02 Hz consisting of episodes of firing that involve both single spikes (1) and HT bursts (2). Bottom traces: after washout of TMA the cell reverts to a pattern of continuous tonic firing (again, see enlarged section below).
(1.82 MB TIF)

Figure S4 Development of the ISO as assessed with extracellular recording and irregularity of the ISO. A. Extracellular single unit recording of a TC neuron in the LGN showing continuous firing in the presence of 50 µM Cch. Additional application of 100 µM *trans*-ACPD converts this into an ISO at ∼0.025 Hz through the appearance of rhythmic pauses in firing. B. ISO recorded intracellularly in the LGN in the absence of steady injected current (top) and following the injection of a small amount of steady hyperpolarizing current (below). Although the ISO is somewhat irregular, the long-lasting hyperpolarizing potentials from which it is sculpted are highly conserved and stereotypical. This is clearly indicated by the averages (black traces) of these potentials which are shown below for the supra- (1) and subthreshold (2) case (the grey traces show the individual events used for constructing the average). Note again the biphasic nature of these events (as indicated by the arrows) (10 µM CNQX and 100 µM APV were present for the recording shown in B).
(1.82 MB TIF)

Figure S5 The complex subthreshold manifestation of the ISO in individual neurons is unaffected by antagonists of ionotropic glutamate receptors. A. Top traces: ISO at ∼0.05 Hz recorded intracellularly in an LGN TC. The underlined section is enlarged below and shows the stereotypical, biphasic nature of the constituent hyperpolarizing events. Bottom traces: these potentials, and therefore the ISO, are unaffected by 10 µM CNQX and 100 µM APV. B. Subthreshold manifestation of the ISO in an LGN TC neuron recorded in the presence of 10 µM CNQX and 100 µM APV. Shown below is an average of the stereotypical hyperpolarizing potentials which make up the ISO which reveals a consistent but brief depolarizing event occurring just prior to their onset. C. ISO at ∼0.025 Hz recorded intracellularly from an LGN TC neuron in the presence of NBQX and APV exhibiting faster depolarizing events during the rising phase (green arrows) demonstrating that they are not dependent on ionotropic glutamate receptors.
(1.87 MB TIF)
