# Supplementary figures and images for: ATP-Dependent Infra-Slow (<0.1 Hz) Oscillations in Thalamic Networks
Source: PLoS One. 2009 Feb 12;4(2):e4447. doi: 10.1371/journal.pone.0004447 (PMC2637539; doi:10.1371/journal.pone.0004447)

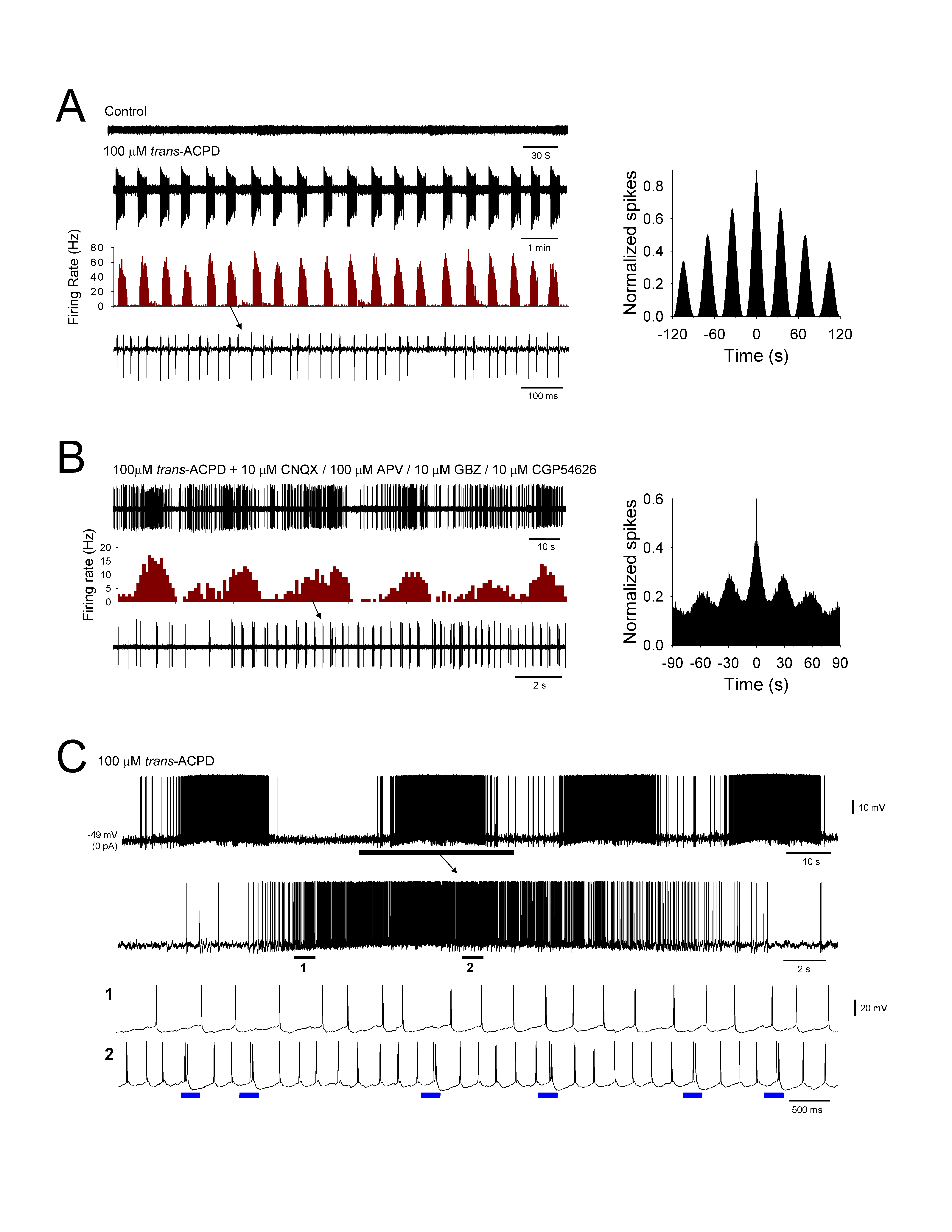

Supplement: Figure S1 — Additional examples of the ISO recorded in the presence of 100 µM trans-ACPD. A. Extracellular recording in a cat LGN slice showing a lack of activity in control conditions (top trace). Application of 100 µM trans-ACPD induces spontaneous firing in a TC neuron that is modulated by an ISO at ∼0.025 Hz (second trace from top). The corresponding firing rate histogram is shown immediately below. Shown further below is an enlarged section from one of the firing episodes, as indicated, which consists of single spike activity only. The corresponding auto-correlogram is shown to the right. B. ISO at ∼0.035 Hz recorded from a TC neuron in a cat VB slice in the presence 100 µM trans-ACPD, 10 µM CNQX, 100 µM APV, 10 µM SR95531 and 10 µM CGP54626 showing episodes of waxing and waning HT bursting. Again, the corresponding firing rate histogram is shown immediately below and an enlarged section of HT bursting shown further below as indicated. The corresponding auto-correlogram is shown to the right. C. Intracellular recording of an LGN TC neuron in the presence of 100 µM trans-ACPD exhibiting an ISO at ∼0.075 Hz. The underlined section is enlarged below. Shown further below are additional enlarged sections (1 and 2) which reveal a mixture of tonic firing and HT bursts (blue bars) (cf. Fig. 1A and C). (2.13 MB TIF) [file pone.0004447.s001.tif]

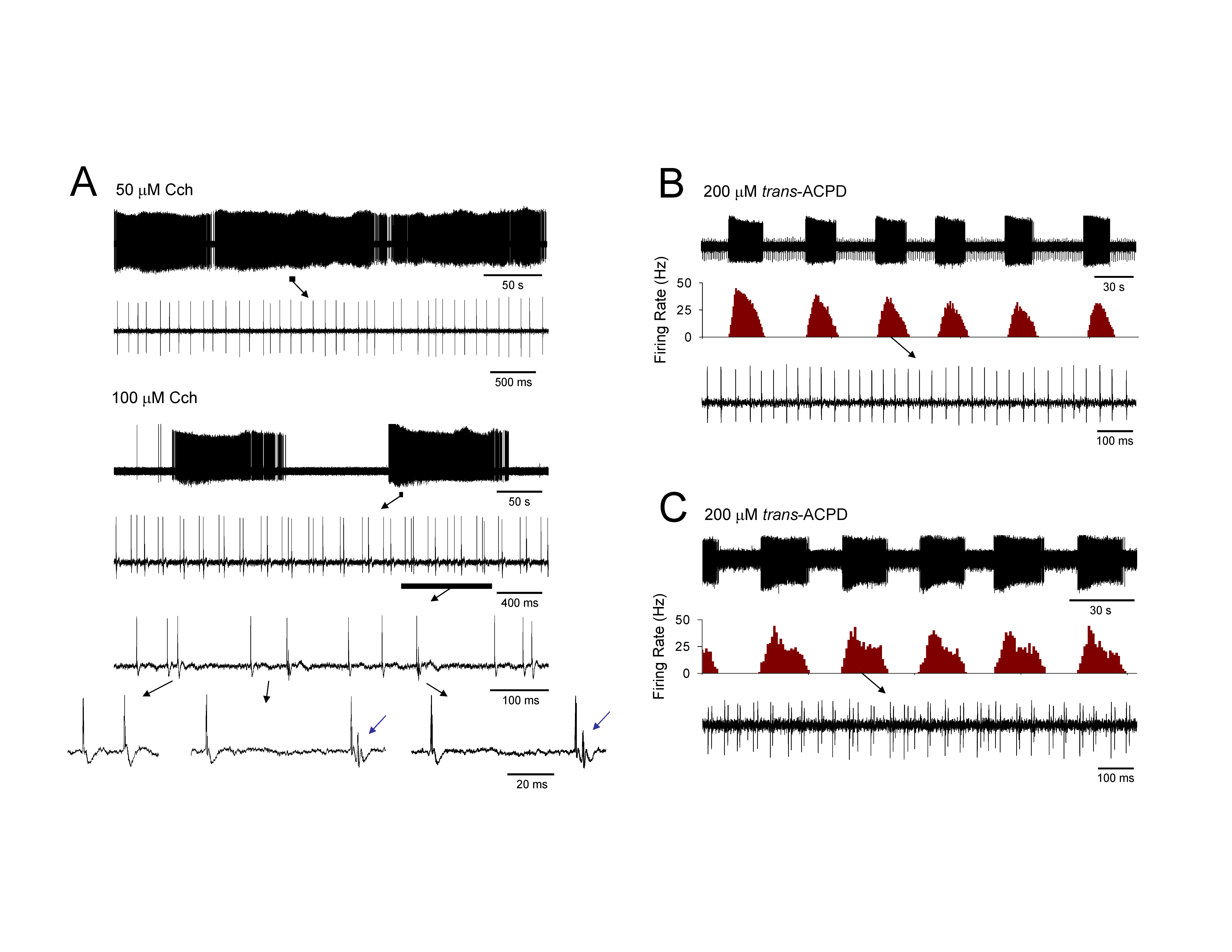

Supplement: Figure S2 — Additional examples of the ISO following intense activation of AchRs or mGluRs. A. Top: irregular, continuous tonic firing recorded in an LGN TC neuron in the presence of 50 µM Cch (see underlined section enlarged below). Bottom: increasing the concentration of Cch to 100 µM converts this activity into an ISO at ∼0.006 Hz which includes periods of HT bursting (see underlined section is enlarged below). Furthermore, these bursts now appear to occasionally drive synchronous activity in additional cells (see blue arrows in bottom traces). B. ISO at ∼0.015 Hz recorded extracellularly from a TC neuron in the LGN in the presence of 200 µM trans-ACPD (note the presence of an additional smaller unit which does not exhibit an ISO). The corresponding firing rate histogram is shown immediately below. Shown further below is an enlarged section from one of the firing episodes, as indicated, which consists of single spike activity only. C. ISO at ∼0.025 Hz also recorded extracellularly from a TC neuron in the LGN in the presence of 200 µM trans-ACPD. The corresponding firing rate histogram is shown immediately below. Shown further below is an enlarged section from one of the firing episodes, as indicated, which in this case comprises HT bursting. (1.66 MB TIF) [file pone.0004447.s002.tif]

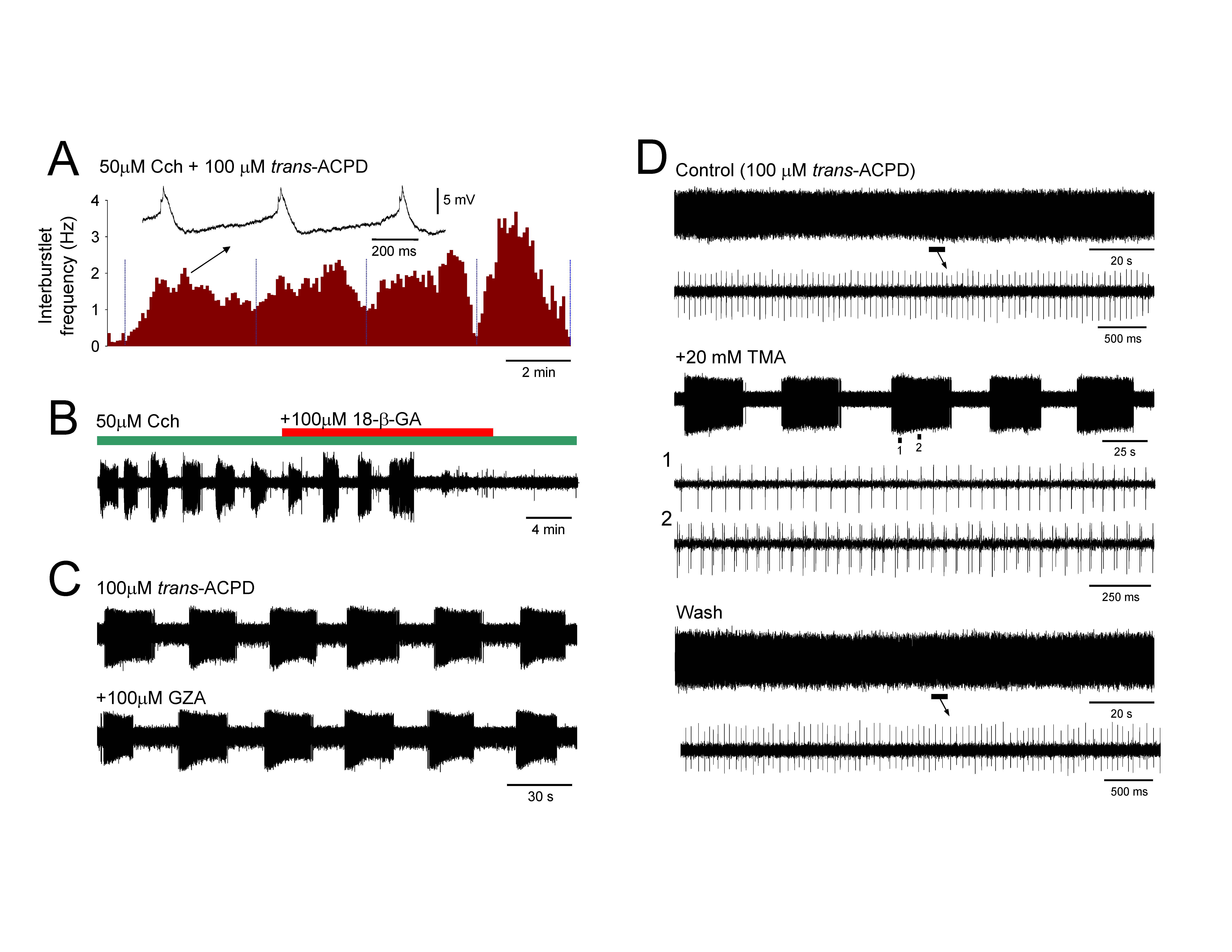

Supplement: Figure S3 — Additional evidence of a role for GJs in the manifestation of the ISO in individual TC neurons. A. Histogram showing the frequency of burstlets over a 14 minute period in an LGN TC neuron recorded intracellularly. Note how this frequency is modulated by an ISO with a frequency of ∼0.006 Hz (blue vertical lines indicate the points of minimum interburstlet frequency). Interestingly, this cell did not otherwise show any overt infra-slow changes in baseline membrane potential. The inset above shows three consecutive burstlets recorded at the point indicated by the arrow. B. ISO at ∼0.006 Hz recorded extracellularly in a VB TC neuron is abolished by application of the GJ blocker, 18β-GA. C. ISO at ∼0.02 Hz recorded extracellularly from an LGN TC neuron in the presence of 100 µM trans-ACPD (top) is unaffected by GZA application (bottom). D. Top traces: LGN TC neuron recorded extracellularly showing continuous tonic firing (see enlarged section below). Middle traces: following addition of 20 mM TMA, the neuron exhibits an ISO at ∼0.02 Hz consisting of episodes of firing that involve both single spikes (1) and HT bursts (2). Bottom traces: after washout of TMA the cell reverts to a pattern of continuous tonic firing (again, see enlarged section below). (1.82 MB TIF) [file pone.0004447.s003.tif]

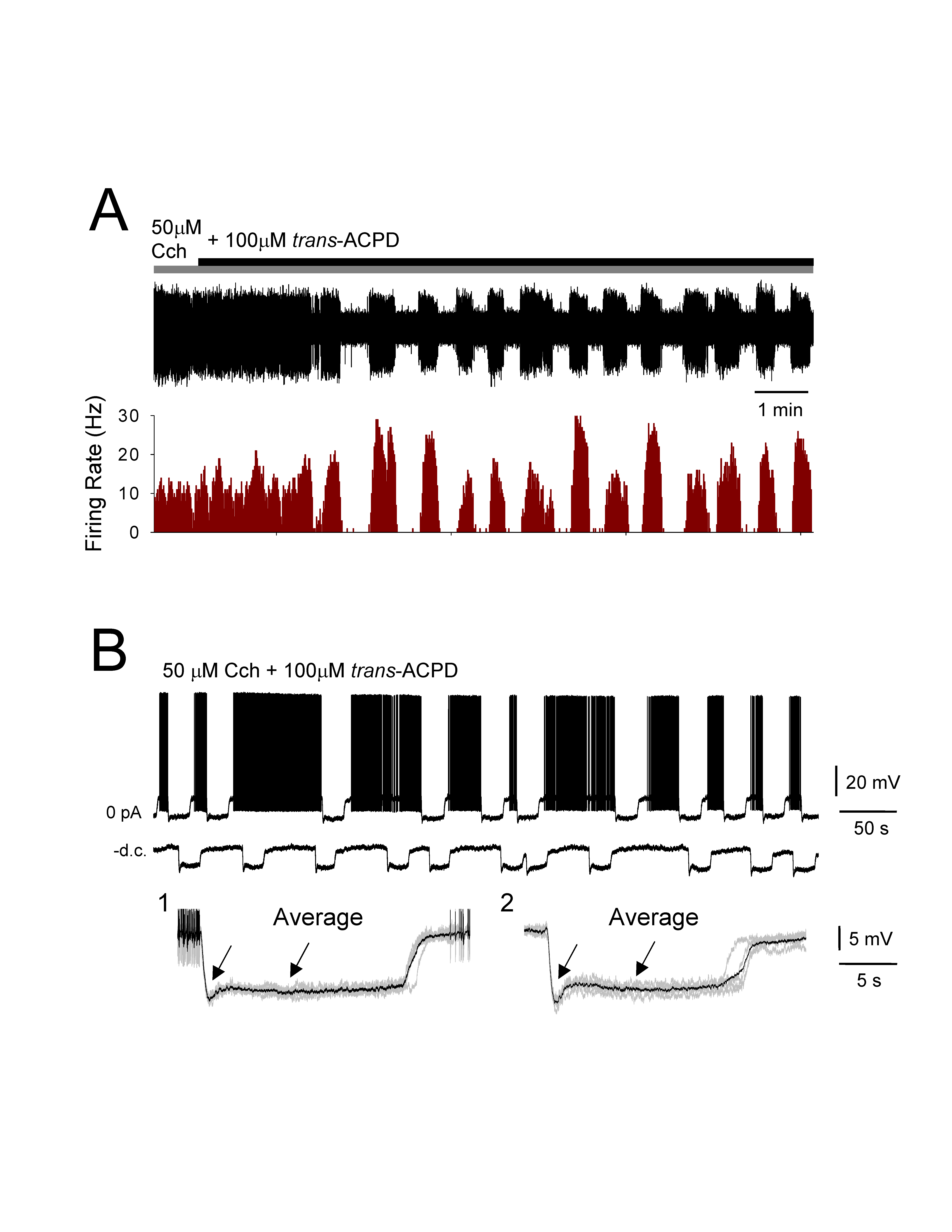

Supplement: Figure S4 — Development of the ISO as assessed with extracellular recording and irregularity of the ISO. A. Extracellular single unit recording of a TC neuron in the LGN showing continuous firing in the presence of 50 µM Cch. Additional application of 100 µM trans-ACPD converts this into an ISO at ∼0.025 Hz through the appearance of rhythmic pauses in firing. B. ISO recorded intracellularly in the LGN in the absence of steady injected current (top) and following the injection of a small amount of steady hyperpolarizing current (below). Although the ISO is somewhat irregular, the long-lasting hyperpolarizing potentials from which it is sculpted are highly conserved and stereotypical. This is clearly indicated by the averages (black traces) of these potentials which are shown below for the supra- (1) and subthreshold (2) case (the grey traces show the individual events used for constructing the average). Note again the biphasic nature of these events (as indicated by the arrows) (10 µM CNQX and 100 µM APV were present for the recording shown in B). (1.82 MB TIF) [file pone.0004447.s004.tif]

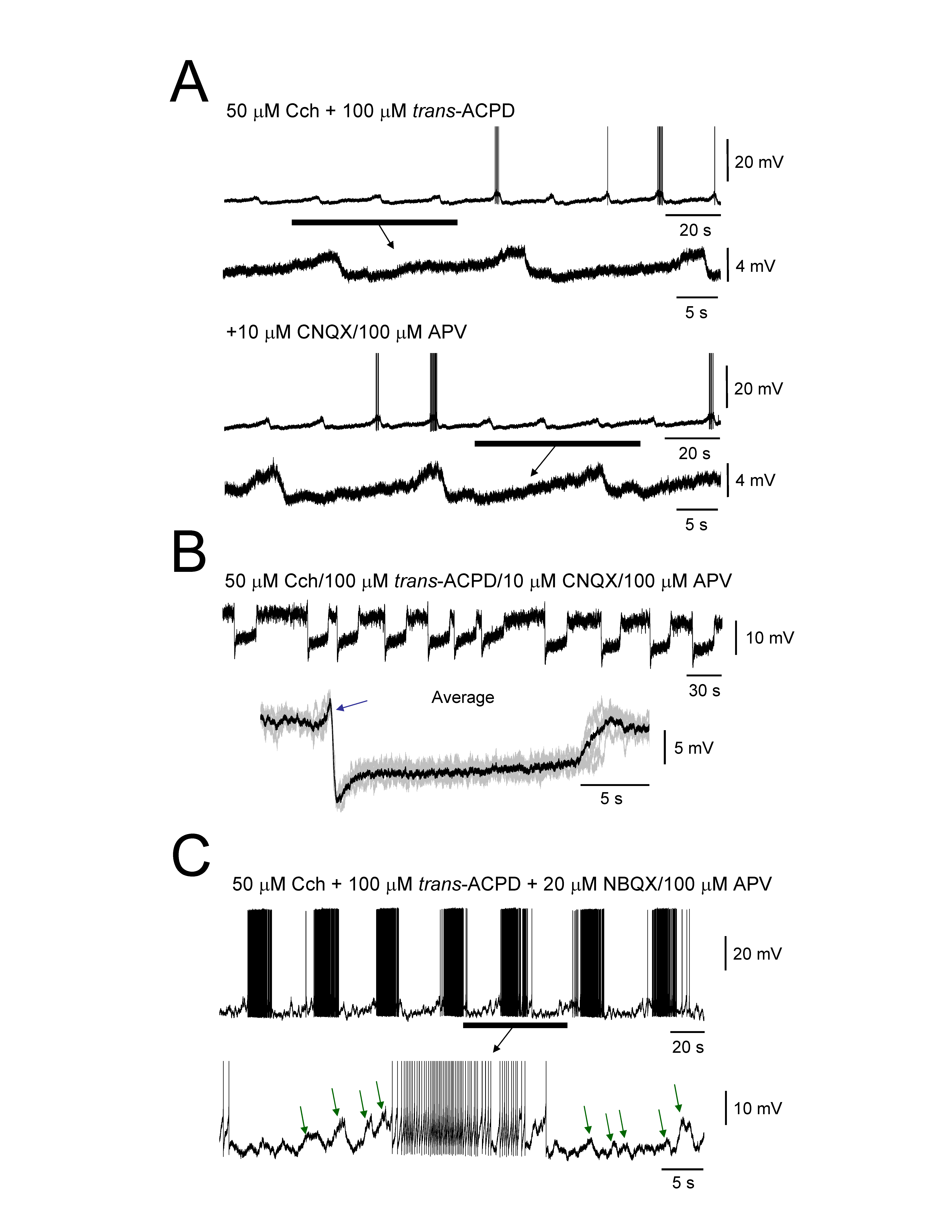

Supplement: Figure S5 — The complex subthreshold manifestation of the ISO in individual neurons is unaffected by antagonists of ionotropic glutamate receptors. A. Top traces: ISO at ∼0.05 Hz recorded intracellularly in an LGN TC. The underlined section is enlarged below and shows the stereotypical, biphasic nature of the constituent hyperpolarizing events. Bottom traces: these potentials, and therefore the ISO, are unaffected by 10 µM CNQX and 100 µM APV. B. Subthreshold manifestation of the ISO in an LGN TC neuron recorded in the presence of 10 µM CNQX and 100 µM APV. Shown below is an average of the stereotypical hyperpolarizing potentials which make up the ISO which reveals a consistent but brief depolarizing event occurring just prior to their onset. C. ISO at ∼0.025 Hz recorded intracellularly from an LGN TC neuron in the presence of NBQX and APV exhibiting faster depolarizing events during the rising phase (green arrows) demonstrating that they are not dependent on ionotropic glutamate receptors. (1.87 MB TIF) [file pone.0004447.s005.tif]
